# U.S. Consumer Perceptions of Food and Pork Production Sustainability: A Cluster-Based Audience Segmentation Analysis

**DOI:** 10.3390/foods15050894

**Published:** 2026-03-05

**Authors:** Kevan W. Lamm, Melvin A. Newell, Alexa J. Lamm, Allison R. Byrd, Fally Masambuka-Kanchewa, Catherine E. Sanders, Shuyang Qu, Michael S. Retallick, Nicholas Gabler

**Affiliations:** 1 Department of Agricultural Leadership, Education & Communication, University of Georgia, Athens, GA 30606, USA; melvin.newell@uga.edu (M.A.N.); alamm@uga.edu (A.J.L.); allisonbyrd@uga.edu (A.R.B.); 2 Agricultural Education and Studies, Iowa State University, Ames, IA 50011, USA; fallymk@iastate.edu (F.M.-K.); squ@iastate.edu (S.Q.); msr@iastate.edu (M.S.R.); 3 Agricultural and Human Sciences, North Carolina State University, Raleigh, NC 27695, USA; ecdobbin@ncsu.edu; 4 Department of Animal Science, Iowa State University, Ames, IA 50011, USA; ngabler@iastate.edu

**Keywords:** sustainable agri-food consumption, consumer perceptions, audience segmentation, cluster analysis, sustainable diets, pork production, sustainability

## Abstract

Despite the importance of a sustainable food system, prior research has not empirically examined whether general food sustainability perceptions and production-specific sustainability perceptions co-occur consistently across audience segments, which is important for informing targeted communication and education strategies. The current research analyzes perceptions from an audience segmentation perspective to inform tailored communication and education strategies. Specifically, a sample of 905 adults in the United States provided perceptions of food production sustainability in general as well as pork production sustainability in particular. Responses were analyzed using a two-step cluster analysis (hierarchical/Ward’s method followed by k-means) based on two sustainability indices reflecting general food sustainability importance and pork-specific sustainability. The results indicate three distinct audience segments with meaningful differences in both general food production and pork production sustainability perceptions: Food Sustainability—First (High/Moderate), Broad Sustainability Advocates (High/High), and Lower Sustainability Salience (Low/Low). Segment differences were further characterized using chi-square tests and multinomial logistic regression, indicating that sex, age category, education, and metro/non-metro status contributed to segment classification, with the Broad Sustainability Advocates segment more likely to include female, metro, and higher-education respondents. The study findings indicate opportunities for segment-specific communication and education to address motivations and barriers and support transitions toward more sustainable agri-food consumption.

## 1. Introduction

The world population is predicted to reach nearly nine billion by 2050, causing a projected 60% increase in the demand for food [1]. With the demand for food rising, consumers and producers must understand the importance of a sustainable food system and how their decisions can affect food system outcomes and markets. In recent decades, agricultural food production has increased drastically to meet population growth while having a direct impact on the environment [2]. Agricultural practices have been criticized as environmentally unsustainable. For example, conventional agricultural practices have been criticized for using large amounts of freshwater, producing around one-quarter of the world’s greenhouse gas emissions, and requiring about half of the world’s habitable land [2]. Simultaneously, climatic events have been linked to decreases in food production, which in turn affects food availability [3,4]. The confluence of these factors has resulted in roughly one in nine individuals “lack[ing] sufficient food to live active and healthy lives” [5] (p. 623), highlighting the need to understand how consumers perceive sustainability in food production and how those perceptions may shape food choices.

The Academy of Nutrition and Dietetics defines a sustainable food system as the ability to “provide nutrition and food security for everyone without compromising the well-being of people or the planet now and in the future” [6] (p. 1). Recent research has found that “consumers are not fully aware of the importance of sustainability; in general, consumers tend to associate sustainable production with just organic farming and higher quality” [7] (p. 1). Additionally, “adults have a positive attitude towards leading a sustainable food choice. However, the concepts and attributes, that define a sustainable diet, are still confusing for most of the population” [8] (p. 10). Together, these findings suggest that sustainability is often interpreted through a narrow set of terms (e.g., “organic” or “quality”), creating a gap between stated intentions and informed decision making. The nexus between perceptions of sustainability and food production is therefore a relevant aspect of the food system, particularly the question of whether general sustainability perceptions translate consistently to specific production contexts, and it is central to efforts aimed at supporting more sustainable agri-food consumption.

The linkage between food production and sustainability is a critical theme related to planetary concerns, as reflected in frameworks such as the United Nations Sustainable Development Goals [9]. Food consumption has one of the largest impacts on the environment [10], and decisions consumers make when purchasing food can shape environmental outcomes through food production practices. However, despite the linkage between consumer choices and subsequent impact, prior research has not empirically examined consumer perceptions of sustainable food production in ways that support actionable, consumer-focused strategies. For example, Fraser et al. [11] noted that dietary habits vary by sex, education, and age, and these consumption patterns can be used to interpret differences among demographic groups and their perceptions of food production and sustainability. The importance of audience differences and nuance is therefore both relevant and central to the issue, which underscores the need to examine perceptions of food production sustainability amongst potential consumers and to identify distinct audience segments to guide targeted communication and education efforts.

Within the food system, the relationship between meat animal production and environmental sustainability has emerged as an important topic [12]. For example, there are numerous studies that have examined the relative environmental footprints of plant- versus animal-based protein (e.g., [13,14,15]). Additionally, studies have examined food consumption trends globally [16], highlighting ongoing shifts in protein demand and consumer choice. The role of pork in diets has surfaced as a global trend, with increased consumption anticipated in the future (e.g., [17]). Therefore, the perception of pork production and sustainability warrants further investigation, particularly given the need to understand how consumers evaluate sustainability across protein sources and how those perceptions may shape consumption decisions. Importantly, prior research has not systematically examined whether consumers who endorse sustainability in general food choices extend those perceptions to specific animal protein production contexts. This distinction is theoretically meaningful, as general sustainability support may not translate to domain (food animal production) perceptions where competing considerations, such as taste preferences, cultural norms, and dietary choices, may attenuate sustainability perceptions. Examining cross-context consistency may provide an empirical basis for better understanding the heterogeneity in sustainability perceptions, which may help to advance beyond descriptive classification to a more theoretically informed perspective of consumer sustainability segments.

### 1.1. Pork Production in the U.S.

The United States is the third-largest pork-producing country in the world, contributing to 11% of global pork production [18]. The U.S. pork industry supports over 600,000 jobs and contributes over $57 billion to the GDP [19], making it one of the largest agricultural contributors to the U.S. economy. Pork was found to be among the most widely consumed proteins around the globe in 2022 [20]. As consumer attention to sustainability increases, pork represents an important case for examining how sustainability perceptions intersect with animal-source food choices. Given the size and scale of the pork production industry in the U.S., many consumers have expressed concerns about its sustainability. For example, a recent study found that about one-third of consumers planned to reduce their pork consumption due to concerns over nutrition, safety, and ethics of raising pork [21]. These concerns span multiple dimensions of sustainability and can shape both purchasing intentions and receptivity to sustainability-related messaging. Consumer interest in sustainable production practices and animal well-being in the pork industry has influenced industry-wide adaptations and consumer communication efforts. For example, a retrospective report found that over the past fifty years, U.S. pork producers have reduced land use per unit by 75.9%, water use by 25.1%, and the potential for global warming by 7.7% [22]. Taken together, the economic significance of pork, ongoing changes in production practices, and persistent consumer concerns underscore the need to segment consumers based on sustainability perceptions related to pork production.

### 1.2. Literature Review

Audience segmentation [23] provides a robust framework within which to review the relationship between consumers and perceptions of sustainability in agricultural production practices, both generally and specifically related to pork production. Segmentation approaches are particularly useful when perceptions are heterogeneous and when “one-size-fits-all” messaging is unlikely to be effective. While audience segmentation using cluster analysis and demographic profiling is well established in the broader sustainability and consumer behavior literature (e.g., [24,25,26]), the present study applies this approach to a specific and previously unexamined question, specifically, whether consumer segments that emerge from the simultaneous consideration of general food sustainability perceptions and pork-specific sustainability perceptions result in meaningful cross-context inconsistencies in sustainability salience. Specifically, segments support the development of tailored communication and education strategies for distinct consumer groups. Previous research has used audience segmentation theory to analyze differences amongst populations related to communication channel preference [24], climate change [25], and public health interventions [26], among others. In the context of sustainable agri-food consumption, segmentation can clarify how different consumers interpret sustainability cues, weigh trade-offs, and respond to sustainability-oriented information, providing an empirical basis for targeted communication and education strategies. This study applies a segmentation approach to consumer perceptions of food production sustainability and pork production sustainability to identify interpretable audience groups and inform more targeted communication and education efforts.

#### 1.2.1. Theoretical Foundations for Cross-Context Sustainability Perceptions

Understanding why consumers may support sustainability in general food choices but may not consistently extend those perceptions to specific production contexts requires engagement with several theoretical perspectives. For example, the value–belief–norm (VBN) framework [27,28] proposes that environmental behavior is shaped by a causal chain from an individual’s values, to their beliefs, to their engagement with proenvironmental personal norms, leading to their behaviors. Within this framework, general sustainability importance may reflect broad value orientations, whereas pork-specific sustainability perceptions may be more related to specific beliefs about consequences and personal responsibility. This is consistent with VBN, where “Environmentally significant behavior depends on a broad range of causal factors, both general and behavior-specific […] Different kinds of environmentally significant behavior have different causes […] each target behavior should be theorized separately” [28] (p. 421).

Additionally, previous research has indicated that there may be an attitude–behavior gap among consumers as it relates to sustainable food consumption [29]. Specifically, consumers who express positive attitudes toward sustainability do not consistently translate those attitudes into product-specific evaluations or purchasing decisions; “Consumers are clearly not a homogenous group, and raising their awareness of the issues involved within food production needs to be targeted accordingly” (p. 186). This pattern suggests that segmenting consumers based on the co-occurrence of general and domain-specific sustainability perceptions may reveal meaningful heterogeneity that a single sustainability measure might miss.

Furthermore, the effects of cognitive dissonance [30] may also be relevant. For example, consumers who support general food production sustainability may experience dissonance when confronted with context-specific sustainability observations which are not compatible with their general beliefs. The tendency would be to dismiss or ignore incompatible data. Therefore, examining both general and pork production independently may help to mitigate the potential to defer to general beliefs. While these mechanisms are not directly tested in the current study, they provide a theoretical basis for anticipating cross-context inconsistency in sustainability perceptions and for interpreting audience segments based on such patterns.

#### 1.2.2. Food Consumption Patterns and Age

Previous research examining the relationship between age and food sustainability has found that “older generations were less aware of sustainability and its related problems” [7] (p. 16). This statement is supported by Franzen and Vogel [31], who found that environmental concerns decreased as age increased. This inverse relationship may be partially explained by the perspective that older individuals may begin to prioritize health over sustainability [7]. Not only does environmental concern decrease with age, but so does the understanding of food sustainability. A recent study found that when “linking sustainability to health, 40% of the participants believed that sustainable diet and healthy diet terms were synonymous” [8] (p. 13). These results suggest that younger individuals may be more aware of sustainability, whereas their older counterparts may lack the appropriate knowledge to make sustainable food choices, highlighting age as a potentially meaningful characteristic for differentiating audience segments.

Food consumption patterns and age have also identified differences between various age groups. For example, Kraus et al. [32] found that “younger consumers attach less importance to naturalness and seem to be more open to high-technology food processing” (p. 124) and that “young men to the greatest extent prefer meat products” (p. 124). Additional research found that “older subjects eat a diet considered more healthful, perhaps out of concern for their deteriorating health” [11] (p. 32). Taken together, prior findings suggest that age may shape both sustainability awareness and the attributes consumers prioritize, which is relevant for interpreting segment differences in sustainability perceptions and consumption-related decision making.

#### 1.2.3. Food Consumption Patterns and Gender

Research examining the role of gender in influencing consumer awareness of food production sustainability is somewhat limited within the academic literature [8]. Nevertheless, one study found that men, compared to women, had a better knowledge of terms relating to food sustainability [8]. However, the same study observed that women were more likely to rank attributes associated with food sustainability as more important compared to their male counterparts. This distinction suggests that knowledge and perceived importance may operate differently across gender groups, which has implications for how sustainability information is framed and communicated. The findings suggest that women may be more aware of, and open to, consuming sustainably produced food than men, indicating that gender may be a useful characteristic for interpreting sustainability-oriented audience segments.

Food consumption patterns between men and women have also been found to have significant differences. Shiferaw et al. [33] found that “a greater proportion of men than women reported eating meat, such as steak and roast, duck and game hen, and ham, whereas more women than men reported eating fruits and vegetables” (p. 454). These findings were consistent with Fraser et al. [11] and Prattala et al. [34], where men were found to consume meat more frequently but fruits and vegetables less frequently compared to women. Together, these patterns suggest gender-linked differences in behaviors, and sustainability-related priorities may help explain variation in perceptions of sustainable food production and associated messaging.

#### 1.2.4. Food Consumption Patterns and Educational Level

Previous research has found a linkage between educational level and food production sustainability choices. Chirilli et al. [35] found that “education level influenced consumers’ awareness of environmental sustainability concepts highlighting that better grades of education bring better information and awareness […] and a higher preference for environmental protection” (p. 16). Educational attainment may therefore function as a proxy for exposure to sustainability information and for the skills needed to interpret complex, and sometimes competing, sustainability claims. The authors speculated that higher education allows for complex topics such as food production sustainability to be brought to an individual’s attention more readily through potential exposure and provides a space for internalization and critical thinking on such topics to occur. This perspective is consistent with Indriani et al. [36], who proposed that “knowledge about environmental problems is an important factor that influences the attitudes of consumers to be more environmentally friendly” (p. 16). These are consistent with Sanchez-Bravo and colleagues’ [7] conclusion that consumers with lower education levels demonstrated the lowest level of knowledge and concern about food production sustainability.

Differences in food consumption based on educational attainment have also been examined within the literature. Kushi et al. [37] found that educational attainment is related positively to eating patterns that may carry a decreased risk of cardiovascular and other chronic disease. In a similar study, participants with a higher level of education ate less meat, more salads, and fewer sweet foods than those with lower levels of education [11]. Additionally, Kraus et al. [32] found that individuals with a lower level of educational attainment felt they had less ability to control their diet and make healthful decisions. Accordingly, education differences may be an important characteristic for sustainability-oriented audience segments and may shape sustainability-related perceptions and behaviors.

#### 1.2.5. Food Consumption Patterns and Metro/Non-Metro Residence

Intra-regional food consumption analysis has also been conducted, specifically regarding the difference between rural and urban living conditions. Residence context may shape food-related norms, access, and information environments, which in turn can influence both consumption patterns and sustainability-related perceptions. Two European studies analyzing food consumption patterns found that rural young people had healthier diets compared to those living in urban areas [38,39]. According to Morris and Northstone [39] “increasing rurality was associated with increases in mean ‘health awareness’ and ‘traditional’ dietary pattern scores” (p. 1438). The study results were consistent with similar studies examining food consumption patterns and rurality. Levin [38] found that “young people from rural areas have a healthier diet than those living in urban areas” (p. 1782). Although these studies were conducted in European contexts, they suggest that rural–urban differences can be associated with variation in dietary patterns and related beliefs. These findings may support the inclusion of metro/non-metro residence as a potentially informative characteristic for developing sustainability-oriented segments.

### 1.3. Study Purpose

The purpose of this study was to examine perceptions of food production sustainability amongst potential consumers in the U.S. using an audience segmentation approach, with a particular attention to whether general food sustainability perceptions and pork-specific sustainability perceptions co-occur consistently or diverge across segments, with the goal of identifying consumer groups that can inform targeted communication and education strategies to support sustainable agri-food consumption.

Because the current study is exploratory in nature, the number and composition of segments are empirically derived rather than specified a priori through hypotheses. The study was guided by research objectives that reflect the expectation that consumer sustainability perceptions may vary meaningfully across general food and domain-specific (pork) contexts and that this variation may be associated with demographic characteristics. These expectations were theoretically grounded based on the value–belief–norm framework, the attitude–behavior gap literature, and cognitive dissonance perspectives. The study was informed by the following research objectives:

1. Identify distinct segments of U.S. consumers based on their perceptions of food production sustainability and pork production sustainability, with attention to whether general food sustainability perceptions and pork-specific sustainability perceptions co-occur consistently or diverge across segments.
2. Describe how consumer segments differ in their sustainability perceptions across the food production and pork production contexts and evaluate the extent to which segments reflect cross-context consistency or inconsistency in sustainability salience.
3. Determine whether consumer segment membership varies across key demographic characteristics (sex, age category, education, and metro/non-metro residence).
4. Assess the relative importance of sex, age category, education, and metro/non-metro residence in differentiating consumer segment membership.

## 2. Materials and Methods

### 2.1. Research Design

To address the research purpose and objective we used a quantitative research design with a cross-sectional survey. Specifically, we were interested in adults in the United States. We developed a sampling frame based on non-probability opt-in sampling methods within the literature (see [40]). Approval was obtained from the University of Georgia Institutional Review Board (IRB #00008098). We established inclusion and recruitment targets according to demographic distributions derived from the U.S. Census to approximate national trends. Because this study relied on non-probability opt-in recruitment, findings are not intended to be interpreted as nationally representative. The data used within the present study were collected as part of a larger research study, and we make this disclosure based on recommendations for clarity within the literature (see [41]).

### 2.2. Instrumentation and Data Collection

The study used two primary constructs: (1) food production sustainability and (2) pork production sustainability. Perceptions of food production sustainability were measured using three items reflecting environmental protection based on the ecological welfare factor proposed by Lindeman and Väänänen [42] commonly used as an extension of the Food Choice Questionnaire (FCQ) developed by Steptoe et al. [43]. The FCQ and adaptations have been used across diverse research contexts, and the ecological welfare items assess general environmental attitudes toward food production that are generally deemed as conceptually applicable across industrialized food systems (e.g., [44,45]) and thus were considered appropriate for use in the U.S. context. Although the broader ecological welfare factor proposed by Lindeman and Väänänen [42] also included a sub-factor associated with animal welfare, the current study only focused on the environmental production dimension. The second construct focused on perceptions of pork production sustainability based on five questions adapted from the Meat Institute [46] to capture pork-related environmental beliefs, concern, and self-reported behavior (e.g., “Pork production has a bigger impact on the environment than plant-based meat product alternatives”). It should be noted that the pork production sustainability index items span multiple construct levels, including self-reported behavior change, general environmental beliefs about pork production, and so forth. Accordingly, the index is interpreted as a composite reflecting pork-related sustainability salience across these dimensions rather than as a unidimensional perceptions measure. Details regarding both scales are provided in Table 1, including items and internal consistency estimates.

To further establish reliability and validity, a confirmatory factor analysis (CFA) was conducted using STATA SE v19 with maximum likelihood estimation. The two-factor CFA model specified a loading of three items on the food production sustainability construct and five items on the pork production sustainability construct. Model fit was assessed based on recommendations and guidelines within the literature (see [47]). The chi-square test was statistically significant (χ^2^(19) = 190.95, *p* < 0.001), which is expected given the sample size (*n* = 905). The Comparative Fit Index (CFI = 0.955) and Standardized Root Mean Square Residual (SRMR = 0.061) both met conventional thresholds for good fit (CFI > 0.95; SRMR < 0.08). The Root Mean Square Error of Approximation (RMSEA = 0.10) was elevated relative to the conventional 0.08 threshold; however, RMSEA is known to be inflated in models with low degrees of freedom and small numbers of indicators per factor [48], and the convergence of acceptable CFI and SRMR values supports the adequacy of the measurement model.

Furthermore, standardized factor loadings ranged from 0.79 to 0.88 for the food production sustainability construct and from 0.65 to 0.85 for the pork production sustainability construct, with all loadings exceeding the 0.50 minimum threshold (see [49]). Composite reliability (CR) was 0.887 for the food production sustainability construct and 0.857 for the pork production sustainability construct, both exceeding the 0.70 threshold [50]. Average variance extracted (AVE) was 0.724 for the food production sustainability construct and 0.546 for the pork production sustainability construct, both exceeding the 0.50 threshold [50], providing evidence of convergent validity. Discriminant validity was assessed by comparing the squared inter-construct correlation (0.325) to the AVE values for each construct; the squared correlation was below both AVE values (0.724 and 0.546), confirming that the two constructs captured distinct variance [51]. CR and AVE values are reported in Table 1.

Respondents also provided a range of demographic characteristics, including age, gender, educational level, state, and zip code. Respondent zip code was used to determine metro or non-metro categorization [52]. Data were collected in May 2024. A total of 1025 responses were obtained. Cases with incomplete data on the clustering variables (n = 120) were excluded, resulting in a sample of 905 cases with complete data for analysis.

### 2.3. Demographics of Respondents

Respondents ranged in age from 19 to 93 (*M* = 49.87, *SD* = 18.59). Respondents were relatively evenly distributed between male and female, most had at least some college education, and most lived in metro areas. Full demographic profiles for the full sample can be seen in Table 2.

### 2.4. Data Analysis

A two-step hierarchical and k-means cluster analysis was used to identify distinct groups of U.S. consumers based on two sustainability indices reflecting (1) general food sustainability importance and (2) pork-specific sustainability beliefs, concern, and behavior-oriented self-reports. Cluster analysis is a data reduction technique that organizes responses into smaller, maximally dissimilar groups (clusters) based on response patterns [53,54,55,56].

Cluster analysis was conducted in IBM SPSS Statistics (v31) using two composite indices: the food production sustainability index and the pork production sustainability index described previously. Prior to clustering, the association between the two indices was examined to ensure multicollinearity was not problematic [57]. Because both indices were computed from items on a common five-point Likert-type response scale, variables were not standardized prior to clustering. Retaining unstandardized scores was preferred as it preserved the original metric of the response scale, resulting in direct interpretability of agreement levels reflected in the index items. However, as a sensitivity check, standardizing the indices did not change the selected cluster solution in terms of interpretability and overall separation. Additionally, the 8 constituent items were also analyzed as a sensitivity check relative to the two index scores. No change in selected clusters was observed.

**Figure 1:**
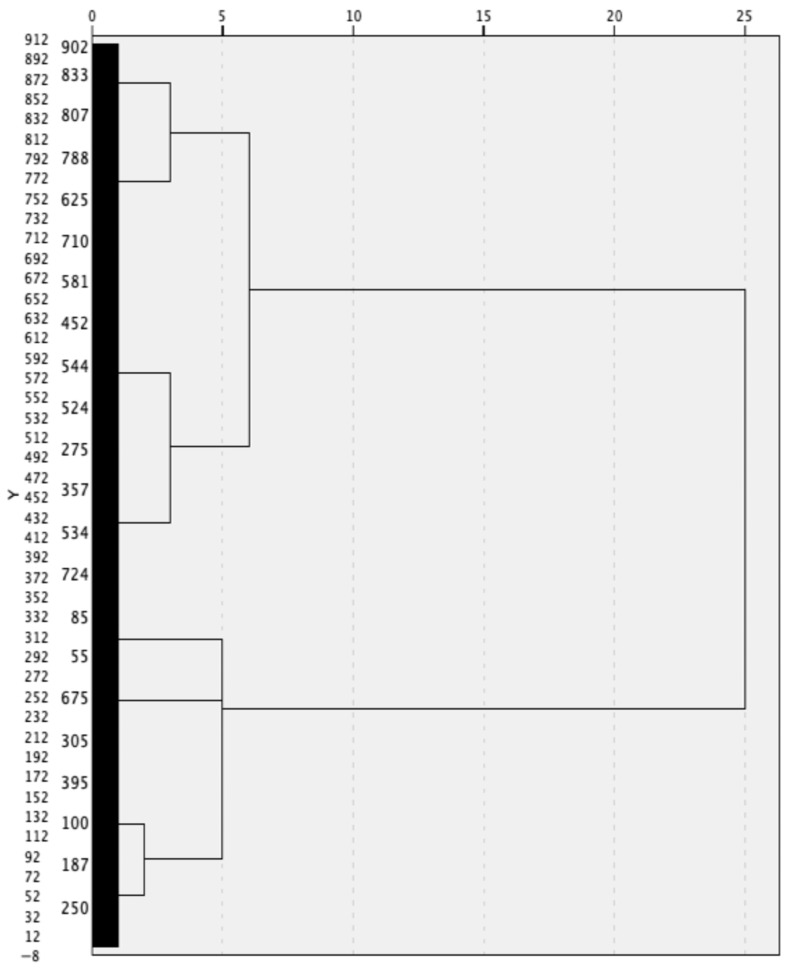
Dendrogram output using Ward linkage to identify number of clusters.

A hierarchical cluster analysis was first conducted using Ward’s method with Squared Euclidean Distance to determine the number of clusters that maximized dissimilarity among resulting groups [56]. The agglomeration schedule and dendrogram (Figure 1) were evaluated, based on preliminary analysis, and a three-cluster solution was initially identified. K-means clustering was then conducted with the number of clusters fixed at k = 3 to refine cluster centers and assign cases to clusters. The k-means algorithm converged at iteration 12. Final segment membership was then saved. Following preliminary analysis, additional internal validation metrics were computed for 2-, 3-, 4-, and 5-cluster solutions to provide an empirical basis for cluster selection. Silhouette coefficients (see [58]) were computed to assess cluster cohesion and separation, and Calinski–Harabasz indices (see [59]) were computed to evaluate the ratio of between-cluster to within-cluster variance across solutions. Additionally, the percentage change in the agglomeration coefficient at each stage was computed to identify the point at which further merging produced a disproportionate increase in within-cluster variance (see [56]). Furthermore, split-sample stability (see [60]) was evaluated by randomly dividing the sample into two approximately equal subsamples and independently applying the two-step clustering procedure. This process was conducted twice with different random splits, yielding four subsamples (n range: 426–479). The basic profile structure was compared across all subsamples to assess whether the same conceptual segment patterns emerged independently [60].

To support interpretation, cluster profiles were summarized using the two composite indices. Differences in index means across clusters were evaluated using one-way ANOVA to describe the magnitude of separation among segments on the clustering indices. Because ANOVA compares clusters on the same variables used to form them, the results are intended as descriptive summaries of the degree of separation achieved by the clustering procedure rather than as independent evidence of cluster validity. Segment differences in categorical demographic characteristics (e.g., sex, age category, education, and metro/non-metro status) were examined using chi-square analyses with effect sizes summarized using Cramer’s V. Finally, multinomial logistic regression was used to examine the unique contribution of demographic characteristics to segment membership, with cluster as the dependent variable and age category, sex, education, and metro/non-metro status entered as predictors (cases with missing predictor data were excluded listwise). Regression results were reported as odds ratios with 95% confidence intervals.

## 3. Results

Initial internal validation metrics were computed for the two-, three-, four-, and five-cluster solutions. Silhouette coefficients indicated weak-to-moderate structure [61] across all solutions (k = 2: 0.416; k = 3: 0.336; k = 4: 0.376; k = 5: 0.344), which is consistent with clustering on less well defined concepts (such as perception) where “performance deteriorates substantially as cluster impablance increases, especially in sparse networks” [62]. To supplement these findings, the Calinski–Harabasz index was calculated. The highest observation was for the two-cluster solution (836.13), followed by the four-cluster (827.62), three-cluster (740.52), and five-cluster (697.37) solutions. However, examination of the change in the agglomeration coefficient (from 770.16 to 947.91; Δ = 177.75) indicated that the largest increase occurred at the merge from three to two clusters, substantially exceeding the increases observed at other stages, indicating that the three-cluster solution represents the point at which further merging produces a disproportionate loss of between-cluster differentiation.

Following preliminary empirical analysis, split-sample stability was evaluated by randomly dividing the sample into two approximately equal subsamples and independently re-applying the two-step clustering process. This process was conducted twice with two different random splits. Across all four resulting subsamples (n range: 426–479), a three-cluster solution emerged with the same general profile structure: a segment endorsing sustainability highly across both indices, a segment with lower endorsement on both, and a segment exhibiting higher general food sustainability endorsement relative to pork-specific sustainability. The replication of this cross-context inconsistency pattern across all subsamples supported the three-cluster framework. Based on split-half analysis, it is important to note that cluster means and distributions varied across subsamples, particularly for the lower-salience segment, which ranged from 14.6% to 31.1%. This variability is consistent with the weak-to-moderate silhouette coefficients and reflects the gradational nature of attitudinal data, where cluster boundaries represent analytic partitions of continuous distributions rather than discrete population groups [61,62]. Based on these findings, the cluster profiles should be interpreted as describing broad, replicable patterns of sustainability salience rather than sharply bounded categories. Overall, the three-cluster solution was retained based on the convergence of the agglomeration evidence, conceptual interpretability of the resulting profiles, and the replication of the qualitative profile structure across subsamples. This multi-criteria approach to cluster selection is consistent with recommendations in the cluster analysis literature, where “In most real life clustering situations, an applied researcher is faced with the dilemma of selecting the number of clusters […when] virtually all clustering procedures provide little if any information as to the number of clusters present in the data” [63] (p. 159).

Based on the empirical analysis, three distinct audience segments emerged through the cluster analysis (see Table 3). The audience segments (identified as clusters in the analysis) were clearly differentiated in their levels of food sustainability perceptions (F = 815.35, *p* < 0.001, η^2^ = 0.643) and pork production sustainability perceptions (F = 670.27, *p* < 0.001, η^2^ = 0.598). Subsequently, segment labels were assigned to support interpretation: Food Sustainability—First (High/Moderate) (n = 363), Broad Sustainability Advocates (High/High) (n = 276), and Lower Sustainability Salience (Low/Low) (n = 266). These labels were developed and intended to reflect relative positions on the measured sustainability indices and should be interpreted as describing perceptions and behavior-oriented self-reports rather than as characterizations of general interest or dispositional orientation toward sustainability in absolute terms. The Broad Sustainability Advocates segment, which exhibited the highest levels of sustainability endorsement across both constructs, reflected the strongest sustainability salience in both general food choices and pork-related contexts. Differences in construct levels across the three segments are presented in Table 3.

Significant differences were identified between the three segments when respondent characteristics were analyzed. Education differed significantly across segments (χ^2^(10) = 26.65, *p* = 0.003), with respondents reporting higher levels of education more prevalent in the Broad Sustainability Advocates segment. In particular, respondents with a graduate or professional degree were most prevalent in the Broad Sustainability Advocates segment (18.8%) relative to the Food Sustainability—First (10.2%) and Lower Sustainability Salience (7.9%) segments. Age category differed significantly across segments (χ^2^(12) = 26.64, *p* = 0.009), with the Broad Sustainability Advocates segment including a larger proportion of respondents aged 30–39 (26.4%) relative to the Food Sustainability—First (15.7%) and Lower Sustainability Salience (16.5%) segments. Metro/non-metro residence also differed significantly by segment (χ^2^(2) = 9.64, *p* = 0.008), with Broad Sustainability Advocates more likely to be metro residents (91.1%) than the Food Sustainability—First (83.6%) and Lower Sustainability Salience (82.7%) segments. Effect sizes were small (Cramer’s V = 0.12 for education; 0.12 for age; and 0.10 for metro status), indicating modest demographic differentiation. Sex differences were not statistically significantly different (χ^2^(2) = 4.03, *p* = 0.133). Distributions of respondent characteristics within each segment are presented in Table 4.

To examine whether demographic characteristics uniquely predicted segment membership when considered simultaneously, a multinomial logistic regression model was estimated with segment membership as the dependent variable (reference segment was Lower Sustainability Salience) and age category, sex, education, and metro/non-metro status entered as predictors. The model improved fit relative to the intercept-only model (likelihood ratio χ^2^(26) = 69.65, *p* < 0.001) and explained a modest proportion of variance in segment membership (Nagelkerke’s R^2^ = 0.085). It is important to note that although the Nagelkerke R^2^ indicates that demographic characteristics account for a limited proportion of variance in segment membership, the model provides a useful descriptive framework for characterizing the demographic composition of the segments. Age category (χ^2^(12) = 30.04, *p* = 0.003), sex (χ^2^(2) = 9.76, *p* = 0.008), education (χ^2^(10) = 28.71, *p* = 0.001), and metro status (χ^2^(2) = 8.52, *p* = 0.014) each contributed to differentiating segment membership in a statistically significant manner. Of note is the fact that sex differences were not statistically significant in the bivariate chi-square analysis (*p* = 0.133) but emerged as a significant predictor in the multinomial model (*p* = 0.008). This pattern may reflect the increased statistical precision in the multivariable models, where controlling for age, education, and metro status differentiates the unique contribution of sex. Nevertheless, this effect should be interpreted cautiously and in the context of the multivariable model rather than as an independent finding.

Relative to the Lower Sustainability Salience segment, respondents were more likely to be classified as Broad Sustainability Advocates if they were female (OR = 1.81, 95% CI [1.24, 2.62], *p* < 0.001), metro (OR = 2.09, 95% CI [1.21, 3.60], *p* = 0.008), and aged 30–39 compared with those aged 70 and over (OR = 2.19, 95% CI [1.22, 3.95], *p* = 0.009). Education also differentiated classification into the Broad Sustainability Advocates segment, with respondents reporting lower education levels less likely to be classified as Broad Sustainability Advocates relative to those holding a graduate/professional degree (ORs = 0.25–0.43, *p*s ≤ 0.012). No individual predictors reached statistical significance for classification into the Food Sustainability—First segment relative to the Lower Sustainability Salience segment, although the overall effects tests indicated that demographic characteristics contributed to segment prediction. Table 5 presents the Broad Sustainability Advocates vs. Lower Sustainability Salience comparison. The Food Sustainability—First vs. Lower Sustainability Salience comparison was also estimated but is not shown for brevity because no individual predictors reached statistical significance.

## 4. Discussion

Environmental sustainability is a growing concern for many U.S. consumers, focusing attention on sustainability practices within agri-food production systems and influencing how consumers evaluate food choices [2]. Due to the concern over potentially unsustainable practices in food production systems, many consumers have also expressed concerns regarding the suitability of animal-based protein in the food system, including how animal-based foods align with environmental sustainability goals [64]. As one of the most consumed animal proteins globally, pork represents a significant contributor to the overall food system [18,19,20]; therefore, perceptions of pork production sustainability represent a noteworthy aspect of the food system more generally, particularly as consumers weigh sustainability trade-offs across protein sources. Empirically examining audience segments’ perceptions provides a unique lens through which to consider broader U.S. consumer trends, as sustainability beliefs and salience are unlikely to be uniform across consumers. This study sought to analyze U.S. consumers’ attitudes towards food production sustainability and pork production sustainability and examine how these attitudes differed between certain clusters based on audience segmentation [23] to inform more targeted sustainability communication and education efforts aligned with sustainable agri-food consumption. The segmentation approach used in this study is exploratory in nature; the resulting segments are empirically derived from the data and should be interpreted as a descriptive framework for characterizing heterogeneity in sustainability perceptions rather than as a confirmatory test of a theoretical model.

Cluster analysis of two sustainability indices (food production sustainability and pork production sustainability) identified three distinct audience segments with interpretable and theoretically coherent profiles spanning (1) general food sustainability importance and (2) pork-specific environmental beliefs, concern, and self-reported behavior. The resulting segments were labeled Food Sustainability—First, Broad Sustainability Advocates, and Lower Sustainability Salience to reflect their pattern of responses across food and pork-related sustainability items.

The Food Sustainability—First segment expressed high endorsement of environmentally friendly food preparation, production, and packaging but only moderate endorsement of the pork production sustainability index, a pattern that is consistent with cross-context inconsistency in sustainability salience. However, because the study did not include direct measures of pork-specific attitudes, consumption frequency, or individual values, this interpretation should be considered an inference from the observed index scores rather than a directly measured finding. In contrast, the Broad Sustainability Advocates segment reported consistently high endorsement across both general food sustainability items and pork-specific sustainability items, including higher overall pork production sustainability scores, suggesting stronger perceived environmental impacts of pork production and stronger personal relevance. Finally, the Lower Sustainability Salience segment reported comparatively low endorsement across both general food sustainability importance and pork-specific environmental concern and behaviors, indicating low sustainability salience in both general food and pork-related contexts.

Demographic analysis of the segments suggested that differences were generally small in magnitude, but the Broad Sustainability Advocates segment tended to include a higher proportion of respondents with graduate/professional education and metro residence and was more likely to include female respondents and individuals aged 30–39 relative to the reference group. In bivariate comparisons, age category, educational level, and metro status differed significantly across clusters, whereas sex differences were not statistically significantly different. In the multinomial model, age category, sex, educational level, and metro status each contributed significantly to cluster classification. Collectively, these results support the use of the three-segment framework for interpreting heterogeneity in sustainability salience, particularly distinguishing respondents who endorse sustainability in general from those who extend sustainability concerns to pork production and consumption contexts. From an applied perspective, the cluster profiles provide a foundation and guide for tailoring sustainability-oriented communication and education strategies to distinct consumer segments.

### 4.1. Contributions to Theory

Within the existing literature there has been limited research to examine the relationship between audience segmentation characteristics (such as demographics) and perceptions of food and pork production sustainability. The current study extends this line of inquiry by empirically deriving three audience segments based on sustainability perception profiles and then examining demographic composition and predictors of segment membership. Specifically, this approach treats sustainability salience as context-dependent, capturing how general food sustainability and pork-specific sustainability co-occur. Combining segment clustering with multivariable prediction of segment membership, the study also helps clarify which demographic characteristics differentiate segments when considered simultaneously.

The most notable theoretical contribution of the study is the identification of cross-context inconsistency in sustainability perceptions. Specifically, the emergence of the Food Sustainability—First segment, which was characterized by high endorsement of general food sustainability importance but only moderate endorsement of pork-specific sustainability, provides empirical evidence that sustainability salience does not transfer uniformly from general food contexts to domain-specific production contexts. This finding indicates that segment-based approaches to sustainability perception may depend on context beyond general perceptions.

Additionally, the observed clusters are generally consistent with expectations associated with VBN framework, which suggests that consumers may differ in the extent to which general sustainability values activate domain-specific beliefs and behavioral norms. The emergence of a Food Sustainability—First segment is consistent with this expectation. Similarly, the attitude–behavior gap literature would anticipate that some consumers express general sustainability endorsement without extending it to specific consumption domains where competing considerations may be more proximal. However, the current study does not directly measure the psychological mechanisms posited by these theoretical perspectives. A recommendation is for future research to further analyze consumer perceptions regarding food-system-related areas using the VBN framework. This approach may help to establish a more empirical linkage between theory and observations.

At a more functional level, the relationship between food sustainability and educational attainment has been well established in the literature [7,35]. Consistent with prior research, in the current study, educational attainment emerged as the clearest demographic differentiator of sustainability salience. Specifically, the most prominent observation from the study was the relationship between an individual’s educational attainment and perceptions of food and pork production sustainability. Across the three segments, education differentiated segment membership and was most strongly aligned with the Broad Sustainability Advocates profile. Notably, this pattern was observed in both bivariate segment profiles and multivariable results, indicating that education retained explanatory value even when age, sex, and metro status were considered simultaneously. Under both conditions, data from this study indicate that individuals with a graduate or professional degree placed a higher level of importance on sustainable production practices related to their food choices. In general, these individuals had a higher concern for the environmental impact of the food they consumed. This relationship is consistent with findings from Indriana et al. [36], who reported that consumers with more knowledge about environmental concerns were more likely to have environmentally friendly consumption patterns. These results may suggest that educational attainment may serve as a proxy for both exposure to sustainability information and the capacity to evaluate competing sustainability claims.

Age was also observed to be a statistically significant predictor of segment membership in the multivariable model. In the analysis, age differences were modest overall; however, the Broad Sustainability Advocates segment included a higher proportion of respondents aged 30–39, and multivariable results indicated that respondents aged 30–39 were more likely to be classified in this segment relative to the 70+ group. These findings are consistent with previous research that has found that environmental sustainability concerns tend to decrease with age [7,31]. Future research is recommended to examine whether other contextual variables (e.g., information environments, social norms, personal relevance) may better explain age-related observations.

In the segment-based analysis, respondents who self-identified as female were more likely to be classified as Broad Sustainability Advocates. This result is consistent with previous research which also found that females tended to rank food sustainability attributes as more important than did males [8]. The results may reflect other gender-related differences in food-related priorities and consumption patterns [33], which could shape how sustainability considerations translate into attitudes and perceptions. Future research is recommended to further analyze the relationship between sustainability perceptions, intent to consume, and actual consumption patterns.

When respondents were analyzed within the metro versus non-metro groups, metro/non-metro residence differed significantly across the three segments, with Broad Sustainability Advocates more likely to be metro residents than the Food Sustainability—First and Lower Sustainability Salience segments. Although the effect size was small (Cramer’s V = 0.10), metro status also contributed to segment membership in the multivariable model, with metro respondents more likely to be classified as Broad Sustainability Advocates relative to the Lower Sustainability Salience segment. These findings indicate modest location-based differences in sustainability segment membership. Several contextual factors may contribute to the observed relationship between metro residence and higher sustainability salience. For example, metro residents may have greater access to specialty and sustainably sourced products, which could increase both exposure to and salience of sustainability-related attributes in food purchasing decisions. Additionally, factors such as local beliefs and exposure to standard agricultural production practices may also be contributing factors. Future research is recommended to confirm these observations and to examine more granular residence categories (e.g., urban, suburban, rural) and potential contextual factors that may also contribute to these observations.

Although demographic characteristics were observed to have statistically significant contributions to segment differentiation, the overall explanatory power of the model was modest (Nagelkerke’s R^2^ = 0.085). This finding suggests that other, unmeasured, constructs likely play a more substantial role in sustainability segment differentiation than demographics alone. For example, psychographic characteristics such as beliefs or information processing style may provide more explanatory power. Future research is recommended to more comprehensively explore contributing variables.

From a theoretical standpoint, the present study contributes to the sustainability perception literature by demonstrating that consumer sustainability salience varies across general food and domain-specific production contexts. The dual-index segmentation approach offers a methodological template for examining cross-context consistency in other production domains (e.g., poultry, controlled-environment agriculture, upcycled food). The identification of a cross-context inconsistency pattern where some consumers express high general food sustainability importance but lower pork-specific sustainability salience aligns with theoretical predictions from the VBN framework and the attitude–behavior gap literature and suggests that future research should continue to examine patterns related to general and specific sustainability perceptions rather than relying on composite measures in isolation.

### 4.2. Contributions to Practice

The findings from this study may hold relevance for multiple stakeholders, including food and pork producers, policymakers, and the general population, though the practical implications should be considered directional given the exploratory nature of the segmentation. First, the results of the study indicated that each of the three audience segments expressed different levels of concern for sustainability, with clear differences in magnitude across segments. Notably, the Food Sustainability—First segment expressed high endorsement of general food sustainability importance, while the Broad Sustainability Advocates segment extended this concern consistently to pork-related beliefs and behaviors. Conversely, the Lower Sustainability Salience segment reported comparatively low endorsement across both food and pork sustainability contexts. The magnitude of differences between segments indicates a certain degree of nuance; however, the primary practical implication is that sustainability communication and education should be directed to segment salience rather than treated as uniform across consumers. Additionally, it is important to note that the current study measured self-reported sustainability perceptions but did not directly assess items such as message responsiveness, information-seeking behavior, or willingness to change consumption patterns. Accordingly, the communication recommendations offered should be viewed as theoretically informed starting points for future research rather than empirically validated strategies. Nevertheless, a recommendation is for both food producers in general as well as pork producers to continue efforts to align production practices with sustainable best management practices. Specifically, these efforts may be most effectively paired with audience-centric communication that reinforces general sustainability cues for the Food Sustainability—First segment, pork-specific sustainability information for the Broad Sustainability Advocates segment, and more foundational, low-assumption messaging for the Lower Sustainability Salience segment. Identifying opportunities to improve sustainable production and then communicating these efforts may help to improve consumer awareness and perceptions.

The heterogeneity in sustainability perceptions observed in this study suggests that uniform sustainability communication campaigns may not be equally effective across consumer segments. The identification of a substantial segment (Lower Sustainability Salience) with comparatively low endorsement of both food and pork sustainability may be informative for designing communication initiatives designed to increase baseline sustainability awareness within this group. Additionally, the cross-context inconsistency observed in the Food Sustainability—First segment suggests that consumers who endorse sustainability in general may not automatically extend those perceptions to specific production contexts, a consideration that may be relevant for efforts targeting context-specific sustainability behaviors. Future research is recommended to specifically assess these recommendations to empirically determine the magnitude of potential impact.

Previous research has indicated that there is a growing preference for sustainable products, especially amongst younger consumers. This trend is particularly noteworthy as this segment also represents a large, and growing, proportion of purchasing power within the market [65]. In the current study, age differences were modest at the bivariate level; however, the multivariable results indicated that age category contributed to segment membership, and respondents aged 30–39 were more likely to be classified as Broad Sustainability Advocates relative to respondents aged 70 and older. Specifically, the sustainable nature of food and pork production might be most valued by consumers under 40. This inference should be interpreted cautiously. In the current analysis, this pattern was most apparent for the Broad Sustainability Advocates segment, which included a comparatively larger share of respondents aged 30–39. A recommendation would be for food and pork producers to develop communication messages tailored to this audience by identifying the platforms on which these consumers are active and the types of media they find most appealing.

In the study, females tended to have a higher level of concern than males related to perceptions of sustainable production in the multinominal model. However, in the segment-based analysis, sex differences were modest and not statistically significant in bivariate comparisons. Therefore, females were more likely to be classified as Broad Sustainability Advocates relative to the Lower Sustainability Salience segment. This observation may indicate there is an opportunity to develop customized communication for different audiences regarding sustainability practices. However, given that sex differences were small, these implications should be interpreted as directional. For example, communication focused on sustainability of pork production may resonate more with female consumers, whereas the nutritional value of pork may resonate more with male consumers [34] and could be evaluated through message-testing research.

Another implication from the current research is the potential to identify specific consumer segments, or niches, in which to specifically direct sustainability-related communication messages. The segment solution provides a practical framework for targeting messages based on sustainability salience rather than demographics alone. Similar to the previous research regarding individuals under 40, the current study would also indicate that individuals with graduate or professional degrees are another important audience characteristic. Consistent with this implication, the Broad Sustainability Advocates segment included the highest proportion of respondents with graduate/professional degrees. Additionally, education was a statistically significant predictor of segment membership, even when other demographic predictors were included in the model. This result may indicate that educational attainment may be a useful consideration for identifying audiences more receptive to sustainability-related information. One recommendation is to consider developing communication messages specifically tailored to this group to clearly identify the actions food and pork producers are taking to improve their production sustainability. For example, highlighting how peer-reviewed research has informed the development of food and pork production may be beneficial, as individuals with graduate and professional degrees may be more familiar with, and confident in, the rigorous standards associated with scientific research. Future research should examine testing communication messages framed from an academic research standpoint with this group to measure their trust in the message and perceptions of sustainability practices.

Lastly, metro/non-metro status showed modest but statistically significant differences between segments, with Broad Sustainability Advocates more likely to live in metro areas. Metro status also remained a significant predictor in the multinomial logistic regression, indicating that the association persisted when age, sex, and education were considered simultaneously, although the overall explanatory power of the model was modest. While residence location may be worth investigating further, additional empirical evaluation is needed before more robust metro/non-metro recommendations can be made. For example, the role of agricultural production proximity or rurality in general may be important contextual factors beyond metro/non-metro status.

Overall, the findings from the current study indicate that sustainability perceptions are not a single concept applicable across contexts. The coexistence of three distinct clusters, particularly one which indicates general food sustainability endorsement but rates pork-specific sustainability lower, suggests that some consumers may benefit from communication that makes explicit connections between general sustainability and specific products. However, the mechanisms underlying this cross-context inconsistency were not directly measured and are recommended to examine in future research.

### 4.3. Limitations

While the results of this study are promising, there are several noteworthy limitations which must be addressed. First, the data were collected through the Qualtrics online platform, and data collection was limited to only those individuals with the requisite technology necessary to engage in the process. Additionally, the nature of non-probability opt-in sampling limits the results’ generalizability. Accordingly, the results (segments) should be interpreted as descriptive of this sample rather than as population estimates. An associated recommendation is for future research to include larger and more diverse samples to improve statistical power. Larger samples would also support additional validation of the segment solution (e.g., replication across independent samples or split-sample stability checks) and permit more granular subgroup analyses where cell sizes may be sparse. More statistical power may help to further analyze and replicate the current study’s findings. Future studies could also evaluate segment stability using alternative clustering specifications to assess the three-segment finding. Second, 120 cases were excluded from the analysis due to incomplete data on the clustering variables. Future research should ensure complete data collection across all items to ensure data completeness.

Another limitation is the instrumentation used in the study, as the scales only measure perceptions of food and pork production sustainability within the U.S., not necessarily the relationship between perceptions and purchasing or consumption intentions. Although the use of perception-based scales is well established within the literature, the use and interpretation of results should be done with care. For example, pork production sustainability combined items including general beliefs about environmental impact, personal concern, etc. While the composite index demonstrated acceptable internal consistency and convergent validity, interpretation of results should be done with care. Similarly, the index did not address considerations such as antibiotic use or production conditions, both of which may be related to perceptions of sustainability. Furthermore, food production sustainability focused only on the environmental protection items from the ecological welfare factor [42] and did not include the animal welfare items from the same scale. Again, the results in the current study should be interpreted with care and future research is recommended to include the full scale for completeness.

In the current study, segments were derived from self-reported sustainability importance, beliefs, concern, and behavior-oriented items; however, these indicators do not directly capture observed purchasing or consumption behavior, nor do they establish causal directionality. An associated recommendation would be to include additional measures of food and pork production sustainability perceptions as well as segment validation variables (e.g., purchasing intentions, willingness to pay, or self-reported purchase frequency), and behavior-based indicators of intention (purchasing or consumption) in future studies. In particular, adding external validation variables would strengthen inferences about the practical meaning of segment membership. Future research could also evaluate whether the same three-segment structure emerges when using alternative measurement specifications (e.g., composite indices or latent variables) and whether segment membership predicts downstream outcomes in longitudinal designs. Additionally, communication or message testing experiments may help to establish whether cluster-based approaches affect perceptions of sustainability generally or context-specifically. Empirically analyzing the predictive relationship between segments, perception, and intention may provide more robust findings upon which to make recommendations.

## 5. Conclusions

The present study provides an empirical analysis of food and pork production sustainability perceptions across three sustainability-salience-based consumer segments. The three-cluster solution was supported by multi-criteria cluster validation, including agglomeration schedule analysis, silhouette coefficients, Calinski–Harabasz indices, and split-sample stability testing, and it is interpreted within a theoretically grounded framework, including VBN and the attitude–behavior gap literature. Based on broader sustainability and policy trends [1], sustainable production in the food system has emerged as a top concern for many consumers. Consistent with this broader context, the segment profiles indicated a distinct group of Broad Sustainability Advocates who expressed high endorsement of sustainability across both general food and pork-specific contexts, alongside a Food Sustainability—First segment, which endorsed sustainability strongly in general food choices but less consistently in pork-specific beliefs and behaviors, and a Lower Sustainability Salience segment characterized by comparatively lower endorsement across items. The results of the study provide a foundation upon which food and pork producers may base communication and education strategies. In particular, the segment solution offers a parsimonious framework for tailoring sustainability-related messaging efforts to consumers who differ in the salience and consistency of sustainability concerns. Identifying the priorities and concerns of consumers is important for food system producers to ensure that products align with market expectations. Future research should replicate these segments across independent samples and examine whether segment membership predicts behavioral outcomes such as purchasing intentions and consumption patterns. Additionally, future research should consider additional behavioral or cognitive constructs in addition to demographic variables to develop more comprehensive models of sustainability perception segment membership.

## Figures and Tables

**Table 1:** Survey items used to measure food sustainability and pork sustainability.

	Number of Items	α	AVE	CR	Statements	Source
Food production sustainability ^a^	3	0.89	0.724	0.887	It is important the food I eat on a typical day has been prepared in an environmentally friendly way	Lindeman and Väänänen [42]
					It is important the food I eat on a typical day has been produced in a way which has not shaken the balance of nature	
					It is important the food I eat on a typical day is packaged in an environmentally friendly way	
Pork production sustainability ^a^	5	0.85	0.546	0.857	I am consuming less pork for environmental reasons	Meat Institute [46]
					Pig farming negatively impacts the environment	
					I am becoming more concerned about the environmental impacts of pork production	
					It is important to me the environment is not negatively impacted because of the pork I eat	
					Pork production has a bigger impact on the environment than plant-based meat product alternatives	

Note. ^a^ Scale used 1 = Strongly Disagree; 2 = Disagree; 3 = Neither Agree nor Disagree; 4 = Agree; 5 = Strongly Agree.

**Table 2:** Demographics of respondents (*N* = 905).

	N	%
Sex		
Male	447	49.4
Female	458	50.6
Age category		
Under 20	15	1.7
20–29	134	14.8
30–39	174	19.2
40–49	146	16.1
50–59	104	11.5
60–69	156	17.2
70 and over	176	19.4
Education		
Less than 12th grade	38	4.2
High school diploma	219	24.2
Some college	219	24.2
2-year college degree	119	13.1
4-year college degree	200	22.1
Graduate or professional degree	110	12.2
Metro Status		
Metro	761	84.1
Non-metro	128	14.1

**Table 3:** Respondents’ levels of food and pork sustainability by cluster.

	Food Sustainability—First (High/Moderate) n = 363 M (SD)	Broad Sustainability Advocates (High/High) n = 276 M (SD)	Lower Sustainability Salience (Low/Low) n = 266 M (SD)	F	η^2^
Food Sustainability	4.11 (0.53)	4.41 (0.58)	2.50 (0.69)	815.35 *	0.64
Pork Production Sustainability	2.79 (0.53)	4.12 (0.49)	2.25 (0.81)	670.27 *	0.60

Note: * *p* < 0.001. ANOVA results are reported to summarize the magnitude of separation among segments on the clustering indices.

**Table 4:** Demographic differences based on sustainability segment.

	Food Sustainability—First n = 363 %	Broad Sustainability Advocates n = 276 %	Lower Sustainability Salience n = 266 %	X^2^	Cramer’s V
Sex				4.03	0.07
Male	49.3	47.5	55.6		
Female	50.7	52.5	44.4		
Age category				26.64 *	0.12
Under 20	1.4	1.4	2.3		
20–29	13.8	14.9	16.2		
30–39	15.7	26.4	16.5		
40–49	14.9	17.8	16.2		
50–59	12.4	8.7	13.2		
60–69	16.8	16.3	18.8		
70 and over	25.1	14.5	16.9		
Education				26.65 *	0.12
Less than 12th grade	5.0	2.9	4.5		
High school diploma	28.4	18.5	24.4		
Some college	24.5	24.3	23.7		
2-year college degree	12.7	12.0	15.0		
4-year college degree	19.3	23.6	24.4		
Graduate or professional degree	10.2	18.8	7.9		
Metro Status				9.64 *	0.10
Metro	83.6	91.1	82.7		
Non-metro	16.4	8.9	16.4		

Note: * *p* < 0.05.

**Table 5:** Multinomial logistic regression predicting segment membership: Broad Sustainability Advocates (High/High) vs. Lower Sustainability Salience (Low/Low).

Predictor	OR	95% CI	*p*
Female (vs. male)	1.81	[1.24, 2.62]	0.002
Age 30–39 (vs. 70+)	2.19	[1.22, 3.95]	0.009
Metro (vs. non-metro)	2.09	[1.21, 3.60]	0.008
Less than 12th grade (vs. grad/prof)	0.25	[0.08, 0.74]	0.012
High school graduate (vs. grad/prof)	0.28	[0.15, 0.55]	<0.001
Some college (vs. grad/prof)	0.43	[0.23, 0.82]	0.010
2-year degree (vs. grad/prof)	0.33	[0.16, 0.67]	0.002
4-year degree (vs. grad/prof)	0.37	[0.19, 0.69]	0.002

Note: χ^2^(26) = 69.65, *p* < 0.001; Nagelkerke’s R^2^ = 0.085.

## Data Availability

The datasets presented in this article are not readily available because the data are part of an ongoing study. Requests to access the datasets should be directed to the corresponding author.
